# Active Vibration Control of Piezoelectric Sandwich Plates

**DOI:** 10.3390/ma15113907

**Published:** 2022-05-31

**Authors:** Zhicheng Huang, Yuhang Mao, Anna Dai, Mengna Han, Xingguo Wang, Fulei Chu

**Affiliations:** 1 College of Mechanical and Electrical Engineering, Jingdezhen Ceramic University, Jingdezhen 333001, China; 1920031003@stu.jci.edu.cn (Y.M.); 120040100230@stu.jci.edu.cn (A.D.); 2120035001@stu.jci.edu.cn (M.H.); wangxingguo@jci.edu.cn (X.W.); 2 Department of Mechanical Engineering, Tsinghua University, Beijing 100084, China; chufl@mail.tsinghua.edu.cn

**Keywords:** piezoelectric sandwich structure, vibration active control, Rayleigh-Ritz method, hypothetical modal method, speed feedback control

## Abstract

This paper deals with the active vibration control of piezoelectric sandwich plate. The structure consists of a substrate plate layer sandwiched between two layers of piezoelectric sensor and actuator. Based on laminate theory and constitutive equation of piezoelectric material, the vibration active control dynamic equation of the sandwich structure is established by using hypothetical mode method and Hamilton principle. The Rayleigh-Ritz method is used to solve it. The form of hypothetical solution is used for approximate solution, which is simple and accurate. The method of this paper is verified by several examples. The parametric studies of the sandwich plate structures are carried out. The results show that applying different boundary conditions and piezoelectric patch positions to the structures have a great influence on the natural frequency. When the driving voltage increases, the deflection of the plate structures increase approximately linearly. The active vibration control studies are investigated as well. The results show that within a certain range, the larger the value of the speed feedback coefficient, the better the active control effect. The positions of the piezoelectric patches affect the effectiveness and cost of active control. When the piezoelectric plate is located at the fixed end, the effect and cost of active control are better than that at the midpoint and free end of the plate.

## 1. Introduction

**Figure 1:**
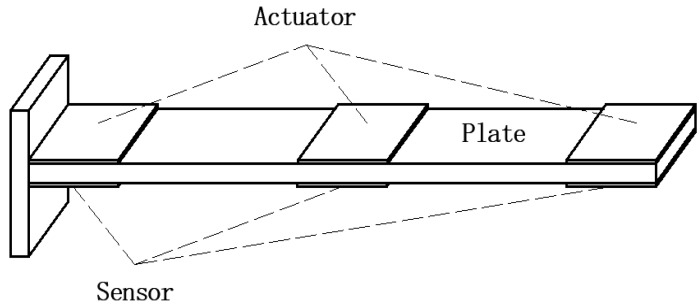
Piezoelectric active control of cantilever plate.

In recent years, due to the widespread use of thin-wall structures in automotive, electronics, aerospace and other fields, the research on its dynamics and active vibration control has gradually emerged [1,2]. Active control technology is often used in engineering to suppress its vibration. The unique piezoelectric effect of piezoelectric materials can realize the conversion of electrical energy and mechanical energy, which is often used as an active control means [3,4,5,6,7]. The typical structure of piezoelectric active control is shown in Figure 1. A substrate plate is sandwiched two piezoelectric material layers [8,9,10]. The positive piezoelectric effect of piezoelectric materials can sense the deformation caused by the excitation of the structure and be used as a sensor. The inverse piezoelectric effect of piezoelectric materials can produce structural changes and suppress vibration, which can be used as actuators.

Since the early 1980s, Swigert and Forward [11] pioneered the active control of piezoelectric structures by using piezoelectric ceramics for vibration control of thin-walled structures. Then Tzou and Gadre [12] derived generalized dynamic equations for piezoelectric actuators and thin polymer shells based on Love’s theory and Hamilton’s principle to suppress their vibrations under actively controlled voltages. Subsequent development of piezoelectric smart structures is diversified and complicated, but they are based on the piezoelectric sandwich composite structure [13]. Various methods are used to model piezoelectric sandwich structures, of which the finite element method is a common method. Jerold et.al [14] investigated the static and dynamic behavior of composite plates with piezoelectric layers symmetrically bonded to the top and bottom surfaces. Sivakumar et.al [15] carried out the static analysis of piezoelectric sandwich cantilever beam with the help of the COMSOL. Prakasha et al. [16] performed a finite element analysis of a thin piezoelectric bimorph with a metal shim using the solid and shell elements, respectively. A formulation of three-dimensional finite element models is proposed to simulate the dynamics of piezoelectric sandwich structures with geometric nonlinearities [17]. Momeni and Fallah [18] proposed a sophisticated mesh free finite volume approach to study the active vibration control of temperature-dependent piezoelectric sandwich composite plates. In addition, some analytical and semi-analytical methods are used for the dynamic analysis of piezoelectric laminated plates. Tanzadeh and Amoushahi [19] developed a finite strip method to analyze the free vibration of piezoelectric laminated plates based on Zigzag theory, refined plate and higher order shear deformation theory. Andakhshideh et al. [20] studied the interlaminar stresses of laminated piezoelectric plates. The electromechanical coupling effect was considered to derive the governing equations, and a three-dimensional multi-term extended Kantorovich method was used to solve it. Gozum et al. [21] presented a semi-analytical model for dynamic analysis of non-uniform plates with piezoelectric patches. Aaa et al. [22] developed a general electromechanical model for modal analysis of a plate with piezo-patches using spectral-Tchebychev technique and Mindlin plate theory assumptions.

In the selection of control law, speed feedback control is a common method for active vibration control of piezoelectric sandwich plates. Li and Narita [23] investigated the relationship between the control gain and active damping ratio of piezoelectric laminated cylindrical panels adopting a velocity feedback control strategy. They numerically calculated the active damping ratio curves and found that the piezoelectric patches are very important for the vibration suppression. Li et al. [24] derived the electromechanical coupling motion equation of piezoelectric fiber reinforced composites With Hamilton’s principle and the Rayleigh-Ritz method. They adopted velocity feedback control rule to obtain active damping for suppression of vibration. Subsequently, Rayleigh-Ritz method was widely used in dynamic modeling of sandwich structures [25,26]. According to the first-order shear deformation theory, based on the virtual displacement principle and Ritz solution, Sfl A et al. [27] deduced the dynamic equation of the piezoelectric laminated cantilever plate. They designed a robust controller using full-dimensional state observer, and compared the control efficiency of the robust controller and speed feedback control with different parameters. Nguyen-Quang et al. [28] studied the dynamic response of carbon nanotube reinforced composite plates integrated with piezoelectric sensors and actuators. The active control of the plate was realized based on a closed-loop velocity feedback control algorithm through the piezoelectric sensors and actuators. Selim et al. [29] studied the free vibration characteristics and active vibration control of composite plates integrated with piezoelectric layers using a meshless approach. A constant velocity feedback controller was used for the active vibration control process of the plates with different positions of piezoelectric sensor and actuator layers. Li et al. [30] deduced the equation of motion for the functionally graded piezoelectric plate based on Hamilton’s principle and Rayleigh-Ritz method. They used a velocity feedback control method to study the effect of external voltage position on active vibration. Wang et al. [31] discussed the control effect of piezoelectric actuator directionality on vibration suppression based on classical velocity feedback control and linear quadratic regulator optimal control.

However, there are relatively few studies on the structural optimization of the piezoelectric sandwich plates. The research on the arrangement of sensors/actuators has gained intensive attention. Shah-Mohammadi-Azar et al. [32] studied the mechanical behavior of a piezoelectric sandwich beam. The effect of electric potential distribution on the electromechanical behavior of the beam was investigated. Nestorović and Trajkov [33] studied the optimal arrangement of actuators and sensors of piezoelectric structures based on balanced reduction of structural models. Araújo and Madeira [34] carried out multiobjective optimization for noise reduction in laminated sandwich plates with surface bonded piezoelectric sensors and actuators. The active damping was implemented by a negative velocity feedback control law. The optimization objectives were to minimize simultaneously the added weight, the number of controllers and noise radiation.

Through reviewing the literatures, it is obvious that the application of piezoelectric materials for active vibration control of thin-plate structures has great potential and deserves more in-depth research. At present, there is little research on the arrangement of sensors/actuators. The numerical optimization analysis of the location arrangement still needs to be continuously explored. The modal dynamics and active control effect corresponding to different position coefficients are still lacking research. In view of the above shortcomings, in the present work of this paper, the dynamic equation of the active control of the piezoelectric sandwich plate is derived based on the laminate theory and the constitutive equation of the piezoelectric structure, combined with the hypothetical mode method and the Hamilton’s principle. The Rayleigh-Ritz method is used to solve the dynamic equation. The method is verified by several numerical examples. Active damping is obtained with speed feedback control. The effects of piezoelectric actuators and sensors at different positions on active vibration control are studied. The control effect with different speed feedback coefficients and control voltages is investigated as well.

## 2. Dynamics Equation of Piezoelectric Sandwich Plates

### 2.1. Fundamental Assumptions

The present analysis is based on the following assumptions:

1. The Kirchhoff assumptions are satisfied, and the midline normal surface is still perpendicular to the elastic surface after the plate is deformed;
2. The bending deformation of the plate is within the elastic range. The expansion and contraction deformation perpendicular to the direction of the plate surface is not considered. The substrate layer and the piezoelectric layer are considered to have the same deflection function;
3. The materials of each layer are firmly pasted, and there is no relative sliding between the layers;
4. The electric field is evenly distributed between the electrodes.

### 2.2. Equation of Motion of the Sandwich Plate under Electric Field

**Figure 2:**
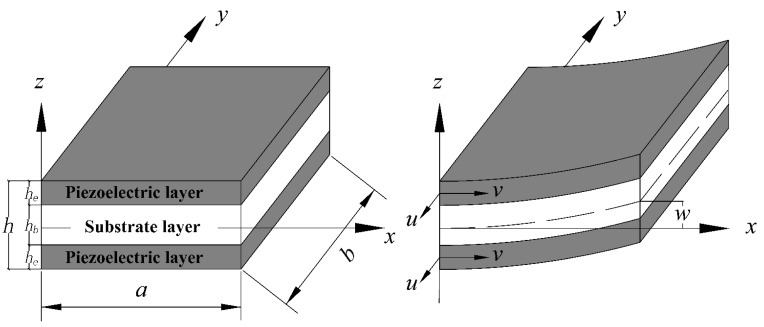
Piezoelectric laminated plate: Before and after deformation.

Consider a simple piezoelectric sandwich plate as shown in Figure 2. The length and width of the plate are *a* and *b*, respectively. The thickness of the substrate and piezoelectric layer are $h_{b}$ and $h_{e}$, respectively.

The rectangular coordinate system is constructed with the middle plane of the plate as the xoy plane. For thin plates, using the classical laminated plate theory the strain–displacement relationships at an arbitrary point (*x*, *y*, *z*) of the piezoelectric laminated plate are given by [30,35]

(1) $$\left[\begin{array}{c} \varepsilon_{x}\\ \varepsilon_{y}\\ \gamma_{xy} \end{array}\right]=\left[\begin{array}{c} -z\frac{\partial^{2}w}{\partial x^{2}}\\ -z\frac{\partial^{2}w}{\partial y^{2}}\\ -2z\frac{\partial^{2}w}{\partial x\partial y} \end{array}\right]$$

where $\varepsilon_{x}$, $\varepsilon_{y}$ and $\gamma_{xy}$ are are the strains in corresponding direction. w is the transverse displacement of the plate. According to the stiffness of the laminate, the transformed stiffness coefficient matrix of the *k*th (*k* = 1, 2, 3) laminate is obtained, and the stress at any point in the structure is obtained according to the generalized Hooke’s law as follows [36]:

(2) $$\left[\begin{array}{c} \sigma_{x}^{k}\\ \sigma_{y}^{k}\\ \tau_{xy}^{k} \end{array}\right]=Q_{k}\varepsilon =\left[\begin{array}{ccc} Q_{11}^{k} & Q_{12}^{k} & Q_{13}^{k}\\ Q_{21}^{k} & Q_{22}^{k} & Q_{23}^{k}\\ Q_{31}^{k} & Q_{32}^{k} & Q_{33}^{k} \end{array}\right]\left[\begin{array}{c} \varepsilon_{x}^{}\\ \varepsilon_{y}^{}\\ \gamma_{{}_{xy}}^{} \end{array}\right]$$

where $Q_{k}$ is the stiffness matrix of the kth layer; $\sigma_{x}^{k}$, $\sigma_{y}^{k}$ and $\tau_{xy}^{k}$ are the stress of the kth layer plate, respectively. $\varepsilon_{x}^{}$, $\varepsilon_{y}^{}$ and $\gamma_{{}_{xy}}^{}$ are the substrate layer (intermediate layer) strains.

The piezoelectric layers are pasted on the two surfaces of the substrate layer, and the constitutive equation for the piezoelectric layer is expressed as follows [37]

(3) $$\left\{\begin{array}{c} \sigma \\ D \end{array}\right\}=\left[\begin{array}{cc} Q_{e} & -e_{e}\\ e_{e}^{T} & {∍}_{i} \end{array}\right]\left\{\begin{array}{c} \varepsilon \\ E \end{array}\right\}$$

where σ and ε are the stress matrix and strain matrix, respectively. $Q_{e}$ is the piezoelectric stiffness coefficient matrix, $e_{e}$ is the constant coupling coefficient of piezoelectric materials, **E** is the electric field strength, **D** is the electrical displacement, and ${∍}_{i}$ is the relative permittivity.

Substitute Equations (1) and (2) and the corresponding piezoelectric constant coefficients into Equation (3), it can be written as,

(4) $$\begin{array}{l} \left\{\begin{array}{c} \sigma_{x}^{k}\\ \sigma_{y}^{k}\\ \sigma_{z}^{k} \end{array}\right\}=\left[\begin{array}{ccc} Q_{11}^{k} & Q_{12}^{k} & Q_{16}^{k}\\ Q_{12}^{k} & Q_{22}^{k} & Q_{26}^{k}\\ Q_{16}^{k} & Q_{26}^{k} & Q_{66}^{k} \end{array}\right]\left\{\begin{array}{c} \varepsilon_{x}\\ \varepsilon_{y}\\ \gamma_{xy} \end{array}\right\}-\left[\begin{array}{ccc} 0 & 0 & e_{31}\\ 0 & 0 & e_{32}\\ 0 & 0 & e_{36} \end{array}\right]\left\{\begin{array}{c} E_{x}\\ E_{y}\\ E_{z} \end{array}\right\}\\ \left\{\begin{array}{c} D_{x}\\ D_{y}\\ D_{z} \end{array}\right\}={\left[\begin{array}{ccc} 0 & 0 & e_{31}\\ 0 & 0 & e_{32}\\ 0 & 0 & e_{36} \end{array}\right]}^{T}\left\{\begin{array}{c} \varepsilon_{x}\\ \varepsilon_{y}\\ \gamma_{xy} \end{array}\right\}+\left[\begin{array}{ccc} {∍}_{11} & {∍}_{12} & 0\\ {∍}_{21} & {∍}_{22} & 0\\ 0 & 0 & {∍}_{66} \end{array}\right]\left\{\begin{array}{c} E_{x}\\ E_{y}\\ E_{z} \end{array}\right\} \end{array}$$

Assuming that the electric field is uniform and the polarization direction of the piezoelectric layer is the *z*-axis direction, that is, there is only a vertical voltage in the *z*-axis direction, then $E_{x}=Ey=0,$ $D_{x}=D_{y}=0$. Substitute them into Equation (4), and the electric field $E_{z}$ and electric displacement $D_{z}$ of the z-direction and be obtained as follows:

(5) $$E_{z}=\frac{V_{0}}{h},\;D_{z}=e_{31}\varepsilon_{x}+e_{32}\varepsilon_{y}+e_{36}\gamma_{z}+\frac{{∍}_{66}}{V_{0}}$$

According to the Rayleigh-Ritz method, the transverse modal displacement at any point on the plate can be expressed as the superposition of modal functions. The form to express the displacement *w* as a generalized coordinate is [38],

(6) $$w\left(x,y,t\right)=\sum_{i=1}^{m}\sum_{j=1}^{n}W_{ij}\left(x,y\right)g_{ij}\left(t\right)=W^{T}{}_{mn}\left(x,y\right)g\left(t\right)$$

where *t* is the time, w(x,y,t) is the modal displacement in generalized coordinates; m and n are the corresponding modal orders, respectively; $W_{mn}\left(x,y\right)$ is the boundary condition of the modal function; g(t) is the generalized coordinate, they are given by,

(7) $$W^{T}\left(x,y\right)=\left[\begin{array}{ccc} W_{11}\left(x,y\right) & \cdots & W_{1n}\left(x,y\right)\\ \vdots & \ddots & \vdots \\ W_{m1}\left(x,y\right) & \cdots & W_{mn}\left(x,y\right) \end{array}\right];\;\;g\left(t\right)=\left[\begin{array}{ccc} g_{11}\left(t\right) & \cdots & g_{1n}\left(t\right)\\ \vdots & \ddots & \vdots \\ g_{m1}\left(t\right) & \cdots & g_{mn}\left(t\right) \end{array}\right]$$

For the sandwich plate system, the shear deformation and moment of inertia are ignored, and the total kinetic energy can be obtained by Hamilton’s principle,

(8) $$\delta \int_{t_{1}}^{t_{2}}\left(T-U+W\right)dt=0$$

where *T*, *U*, *W* are total kinetic energy, total potential energy and external force work, respectively. 

The total kinetic energy is in the following form:

(9) $$\begin{array}{l} T=T_{b}+T_{e}\\ T_{b}=\frac{1}{2}\int_{V_{b}}^{}\rho_{b}{\left(\frac{\partial w}{\partial t}\right)}^{2}dV\\ T_{e}=\frac{1}{2}\int_{V_{e1}+V_{e2}}^{}\rho_{e}{\left(\frac{\partial w}{\partial t}\right)}^{2}dV \end{array}$$

where $T_{b}$ and $T_{e}$ are the kinetic energy of the substrate layer and piezoelectric layer, respectively, $\rho_{b}$ and $\rho_{e}$ are their density, $V_{b}$, $V_{e1}$ and $V_{e2}$ are the volume of the substrate layer, the piezoelectric sensing layer and piezoelectric actuation layer, respectively.

The total potential energy is in the following form:

(10) $$\begin{array}{l} U=U_{b}+U_{e}\\ U_{b}=\frac{1}{2}\int_{V_{b}}^{}{\left\{\varepsilon_{b}\right\}}^{T}\{\sigma_{b}\}dV\\ U_{e}=\frac{1}{2}\int_{V_{e1}+V_{e2}}^{}{\left\{\varepsilon_{b}\right\}}^{T}\{\sigma_{b}\}dV-\frac{1}{2}\int_{V_{e1}+V_{e2}}^{}D_{e1}E_{e1}+D_{e2}E_{e2}dV \end{array}$$

where $U_{b}$ and $U_{e}$ are the potential energy of the substrate layer and piezoelectric layer, respectively, $\varepsilon_{b}$ and $\sigma_{b}$ are the corresponding strain and stress, respectively; $D_{e1}$, $D_{e2}$ and $E_{e1}$, $E_{e2}$ are the electric displacement and electric field strength of the piezoelectric sensing layer and piezoelectric actuation layer, respectively, here $E_{e1}=E_{e1}=E_{z},\;D_{e1}=D_{e1}=D_{z}$.

The virtual work expression of the sandwich plate system is as follows:

(11) $$\delta W=q\int_{A}^{}\delta w\left(x,y,t\right)dA+\int_{A}^{}D_{z}\delta V_{e}dA$$

where the first term is the virtual work of the external force, and the second term is the electric field work. q is the external load function, A is the plate surface area, and $V_{e}$ is the external voltage function.

Substitute Equations (1)–(3) and (6) into Equations (9)–(11), the following energy equation for g(t) can be obtained:

(12) $$T=\frac{1}{2}{\dot{g}}^{T}\left(t\right)\left(M_{b}+M_{e}\right)\dot{g}\left(t\right)$$

(13) $$U=\frac{1}{2}g^{T}\left(t\right)\left(K_{b}+K_{e}\right)g\left(t\right)+V_{e}\left(t\right)K_{c}g\left(t\right)-\frac{1}{2}K_{d}V_{e}^{2}\left(t\right)$$

(14) $$W=Fg\left(t\right)-F_{e}g\left(t\right)V_{e}\left(t\right)+K_{d}V_{e}^{2}\left(t\right)$$

where $K_{b},K_{e}$ and $M_{b},M_{e}$ are the modal stiffness matrix and mass matrix of the substrate and piezoelectric layer, respectively. $K_{c}$ is the electromechanical coupling matrix, $K_{d}$ is the piezoelectric layer capacitance matrix, F is the external force matrix of the plate; F is the force matrix of the substrate, $F_{e}$ is the modal load matrix generated by the piezoelectric layer, and the specific form of the above matrix is given in the Appendix A; $V_{e}\left(t\right)$ is the externally applied active control voltage equation.

Substituting the Equations (12)–(14) into Equation (8), the following equations for g(t) and $V_{e}\left(t\right)$ are obtained:

(15) $$(M_{b}+M_{e})\ddot{g}(t)+K_{{}_{c}}^{T}V_{e}(t)+(K_{b}+K_{e})\;g(t)=F^{T}$$

(16) $$\left(K_{c}+F_{e})\;g(t)=2K_{d}V_{e}(t\right)$$

Equation (15) is the actuation equation, which represents the relationship between the structural deformation caused by externally applied voltage $V_{e}\left(t\right)$; Equation (16) is the sensing equation, which represents the relationship equation between the piezoelectric layer $F_{e}$ and the voltage $V_{e}\left(t\right)$.

If the voltage is eliminated, the static vibration problem can be obtained, which can be described as,

(17) $$\begin{array}{l} Kg(t)=F\\ (K-\omega_{n}{}^{2}M)g(t)=0 \end{array}$$

where $M=M_{b}+M_{e}$ is the total mass matrix, $K=K_{b}+K_{e}$ is the total stiffness matrix, $\omega_{n}$ is the corresponding natural frequency of each order.

### 2.3. Feedback Control Dynamics Equation

The speed feedback control method can be used to provide active vibration control for piezoelectric sandwich plates. In the control law, the velocity change in the amplitude direction of the sensor observation point $\left(x_{0},y_{0}\right)$ is used as the independent variable. The control voltage $V_{e}(t)$ required for the feedback control is the dependent variable. The vibration is controlled by the inverse electric effect of the piezoelectric sheet, and the control voltage is proportional to the speed. The control gain is $G_{\;v}$. Substitute it into the modal Equation (6), and the control voltage can be expressed as:

(18) $$V_{e}(t)=G_{\;v}\dot{w}\left(x,y,t\right)=G_{\;v}W^{T}\left(x_{0},y_{0}\right)\dot{g}\left(t\right)$$

where $\left(x_{0},y_{0}\right)$ is the coordinates of the sensor observation point. Substitute Equation (18) into Equation (15), and the equation of motion with damping can be obtained,

(19) $$M\ddot{g}(t)+G_{\;v}K_{{}_{c}}^{T}W^{T}\left(x_{0},y_{0}\right)\dot{g}\left(t\right)+Kg(t)=F^{T}$$

The active damping matrix is,

(20) $$C=G_{\;v}K_{c}{}^{T}W^{T}\left(x_{0},y_{0}\right)$$

It can be seen from Equation (20) that the active damping is determined by the modal function of the sensor observation point and the speed feedback coefficient. Then the overall kinematics equation is as follows:

(21) $$M\ddot{g}(t)+C\dot{g}\left(t\right)+Kg(t)=F^{T}$$

In the Equation (21), whether the force matrix **F** is constant values or variable with respect to time t, the equation is a second-order constant coefficient inhomogeneous linear equation. The mass matrix **M** and stiffness matrix **K** are diagonal matrices, but the active damping matrix **C** is not, therefore, it cannot be solved directly.

This paper adopts the form of a hypothetical solution, and then continuously approximates to directly decouple [39]. Considering the influence of damping, the modal superposition analysis is used to decouple the equation. One of the pair of linearly independent solutions with respect to the system time response g(t) is,

(22) $$g(t)=\lambda e^{(\alpha }{}^{\pm i\beta )t}$$

After Euler transformation, one acquires,

(23) $$g(t)=\lambda e^{\alpha }{}^{t}\sin\beta x,g(t)=\lambda e^{\alpha }{}^{t}\cos\beta x$$

where λ is the eigenvector of the system; α and β are constant coefficients, and,

(24) $$\alpha +i\beta =\eta_{m_{0}n_{0}}+i\omega_{m_{0}n_{0}}$$

where $i=\sqrt{-1}$; $m_{0\;}$ and $n_{0\;}$ represent the modal orders. According to the Rayleigh-Ritz method, $\eta_{m_{0}n_{0}}$ is the corresponding measured damping value under the freely damped modal system $\left(m_{0},n_{0}\right)$; $\omega_{m_{0}n_{0}}$ is the natural frequency of the modal system $\left(m_{0},n_{0}\right)$.

In order to solve the dynamic Equation (21), the modal function $W_{mn}\left(x,y\right)$ needs to be obtained, which depends on the boundary conditions. At the same time, it is necessary to know the force matrix **F**, which is given in the Appendix A. The load q in the force matrix is the pressure load uniformly distributed on the plate plane, and the harmonic response expression is given by,

(25) $$q=q_{0}\sin\omega t$$

where $q_{0}$ is the excitation constant.

The steady-state solution of **g**(*t*) is obtained,

(26) $$g(t)=A_{mn}\sin\left(\omega t\right)+\;B_{mn}\sin\left(\omega t\right)\;\;\;$$

where $A_{mn}\;\;$ and $B_{mn}\;$ are the corresponding amplitudes in each order mode, which can be obtained by approximating and solving, and then substituting the solution into the dynamic equation, it can be solved.

## 3. Numerical Results and Analytical Investigations

In this section, parametric studies are carried out to study the effects of the position of the piezoelectric patch, the mechanical boundary conditions and the driving voltages. Comparison studies based on three cases are presented to prove the accuracy of the current methodology, which show evident agreement with COMSOL and references. Furthermore, active vibration control studies are carried out to study the active control effect of speed feedback control law on different structures.

### 3.1. Parametric Studies

#### 3.1.1. Case I: The Actuator and Sensor Are Located at Different Locations

A cantilever plate structure with piezoelectric patches at different locations is considered here. Only the influence of the positional configuration of the piezoelectric layer in a single direction (*x*-axis) on the active vibration control is considered. The material parameters of the substrate and piezoelectric patch are shown in Table 1.

**Figure 3:**
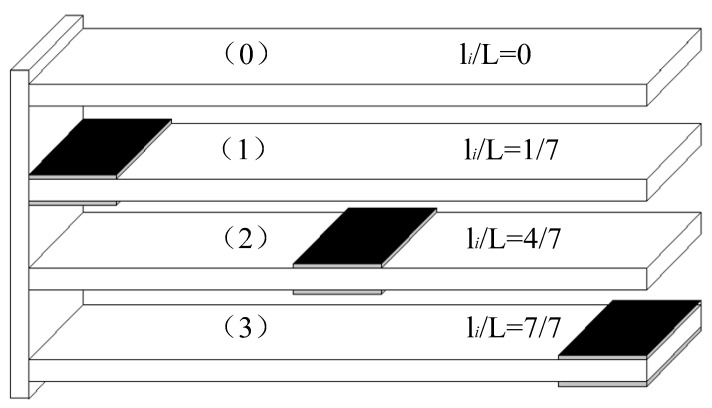
Cantilever plate structures with piezoelectric layers arranged in different positions.

The symmetrically arranged piezoelectric structure does not change the neutral plane of the structure, and is not affected by the tension-bend coupling effect, and has a better numerical matrix of the structure model [22,29,40]. Here the piezoelectric layer and the sensing layer are arranged symmetrically at the top and bottom of the same position. The sensors and actuators pasted on the symmetrical sides of the structure constitute the smallest phase system with maximum stability and robustness. Under the condition that other parameters remain unchanged, piezoelectric layers are arranged at different positions of the substrate as control groups, respectively. The different structures are shown in Figure 3. In Figure 3, $l_{i}/L$ represents the position coefficient along the *x* direction. (0), (1), (2) and (3) represent the codes of each group structure, respectively. $l_{i}/L=0$ indicates that no piezoelectric sheet is arranged. $l_{i}/L=1/7$ indicates that the piezoelectric sheet is arranged at the fixed end. $l_{i}/L=4/7$ indicates that the distance between the piezoelectric sheet and the fixed end is 120 mm. $l_{i}/L=7/7$ indicates that the piezoelectric sheet is arranged at the free end.

The mechanical analysis of the four structures is carried out by the method in this paper. First, the static parameters are solved under the condition of steady-state voltage $V_{e}(t)=0V$ and pulse $q\left(t\right)=0\;N/m^{2}$. For the cantilever structure, the functional form of $W_{mn}\left(x,y\right)$ is the product of the cantilever beam shape function $X_{m}\left(x\right)$ along the *x*-axis and the free beam shape function $Y_{n}(y)$ along the *y*-axis direction [38].

(27) $$\begin{array}{l} W_{mn}\left(x,y\right)=\sum X_{m}(x)Y_{n}(y)\\ X_{m}(x)=\cosh(\eta x)-\cos(\eta x)-\frac{\sinh(\eta l)-\sin(\eta l)}{\cosh(\eta l)+\cos(\eta l)}[\sinh(\eta x)-\sin(\eta x)]\\ Y_{n}(y)=\sinh(\gamma y)+\sin(\gamma y)+\frac{\cos(\gamma l)-\cosh(\gamma l)}{\sin(\gamma l)+\sinh(\gamma l)}[\cos(\gamma y)-\cosh(\gamma y)] \end{array}$$

where η=(π/2)(2m−1),γ=(π/2)(2n+1).

Since the first few modes can relatively completely describe the dynamic characteristics of the structure and affect the effect of active control [41], this paper only discusses the first eight modes.

The calculated first eight natural frequencies of the structure with partially laid piezoelectric patch are compared with the results of the COMSOL. The results are shown in Table 2. Δ% presents the error.

Dynamic characteristics provide the control basis for active control. In this study, the passive damping of the structure has little effect on the natural frequency. However, after arranging the piezoelectric patch at different positions, the natural frequencies change accordingly. As shown in Table 2, with the change of the position coefficient $l_{i}/L$ of the laid piezoelectric layer, the modal parameters of each order change gradually. Under the condition that the overall mass remains unchanged, the change of the position coefficient $l_{i}/L$ leads to the change of the stiffness matrix **K**, and the natural frequency of the structure will change accordingly. With the increase of the position coefficient, the stiffness of the structure decreases under the same mass, resulting in a decrease in the natural frequency. The reduction of the natural frequency will lead to the reduction of the corresponding control frequency. Under the same conditions, a larger mechanical energy needs to be provided to achieve active control.

Among the three structures, structure (3) has the lowest natural frequency, followed by (2), and which of structure (1) is the highest. By comparing the calculation results of the method presented this paper with the COMSOL simulation results, it can be found that they are in good agreement. The minimum error of the first 8 order natural frequencies is 0.6%, the maximum error is 8.5%, and the average error is 2.9%. The calculation results show that the increase of parameters m, n helps to reduce the error, but it will greatly increase the computational cost. Moreover, this paper uses the assumed mode approximation to solve the dynamic equation, so the accuracy is also related to the assumed mode value. In general, the analytical solution in this paper is within the error range, which shows that the model can well describe the vibration characteristics of piezoelectric cantilever plate.

#### 3.1.2. Case II: The Structure with Different Boundary Conditions

Taking the structure 1 in Figure 3 as the research object, the first six-order natural frequency values under different boundary conditions are calculated, and compared with the COMSOL, and the results are listed in Table 3. The boundary conditions of the four sides of the plate are represented by letters, and their meanings are: S-simple support, C-fixed support, F-free. The fixed side of the structure 1 in Figure 3 is defined as the cantilever end, and its opposite side is the free end. CFSF means the cantilever end is fixed, the free end is simply supported and the other two sides are free; SFSF means the cantilever and free ends are simply supported, and the other two sides are free; CFFF means the cantilever end is fixed, and the other three sides are free; CFCF means the cantilever and free ends are fixed, and the other two sides are free.

As can be seen from Table 3, the solution in this paper is in good agreement with the COMSOL solution. The minimum error is 0%, the maximum error is 8.5%, and the average error is 2.8%, which provides verification for the accuracy of the model in this paper. In addition, it can be seen from the above calculation results that applying different boundary conditions to the same structure has a great influence on the natural frequency.

#### 3.1.3. Case III: Deflection Change of Structure under Different Voltages

**Figure 4:**
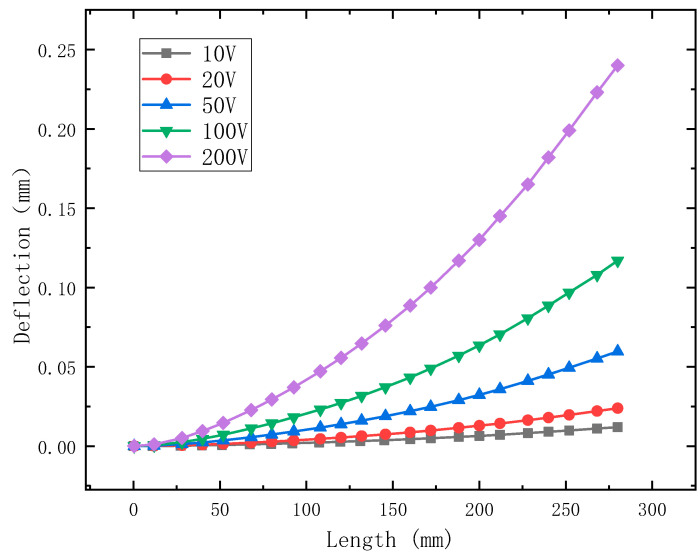
Deflection change of structure (1) under different voltages.

Taking structure (1) as the research object, when it is in a static state, different steady-state voltages are applied to its piezoelectric layer, and the variation of its deflection is obtained as shown in Figure 4. The applied steady-state voltages are **V***_e_*(*t*) = 10 V, 20 V, 50 V, 100 V and 200 V, respectively.

Figure 4 shows the effect of different input voltages on the deflection of the piezoelectric cantilever plate. It can be seen from Figure 4 that when the driving voltage is applied, the cantilever plate will produce corresponding deflection deformation, which is due to the inverse piezoelectric effect of the piezoelectric layer that causes the plate structure to be deflected in the thickness direction. It is observed that when the input voltage becomes larger, the deflection of the plate also becomes larger, as expected. Obviously, the shape of the piezoelectric layer will change under different voltage excitation, which can realize the active control of the vibration of the structure.

In order to further verify the presented method, here consider a bimorph piezoelectric cantilever beam with the geometry, thickness and boundary conditions from reference [42]. When an external electric field is applied to the piezoelectric layer, the cantilever beam produces bending deformation due to the inverse piezoelectric effect. In reference [42], two methods are used to calculate the deflection of the beam end under different voltages. One method is the cell-based smoothed discrete shear gap method (CS-FEM-DSG3), and the other is the discrete shear gap method (DSG3). In reference [43], the analytical solutions of the structure were given as well. The method in this paper is used to calculate the deflection value of the beam end under different voltages, and it is compared with the references. The results are shown in Table 4.

It can be seen from Table 4 that the results by the present method match well with the analytical solution [43]. All errors are less than 0.08%. Compared with the analytical solution, the accuracy of the method in this paper is the highest, followed by DSG3 [42], and the lowest is CS-FEM-DSG3 [42]. In general, the three methods are in good agreement with the analytical method. The results show that the method in this paper has good computational accuracy. Moreover, the effect of different input voltages on the tip deflection of the piezoelectric bimorph beam can be observed. When the input voltage becomes larger, the tip deflection of the beam also becomes larger, which is consistent with the change trend shown in Figure 4.

### 3.2. Active Vibration Control Studies

#### 3.2.1. Case I: Control Effect of Different Speed Feedback Control Coefficient

**Figure 5:**
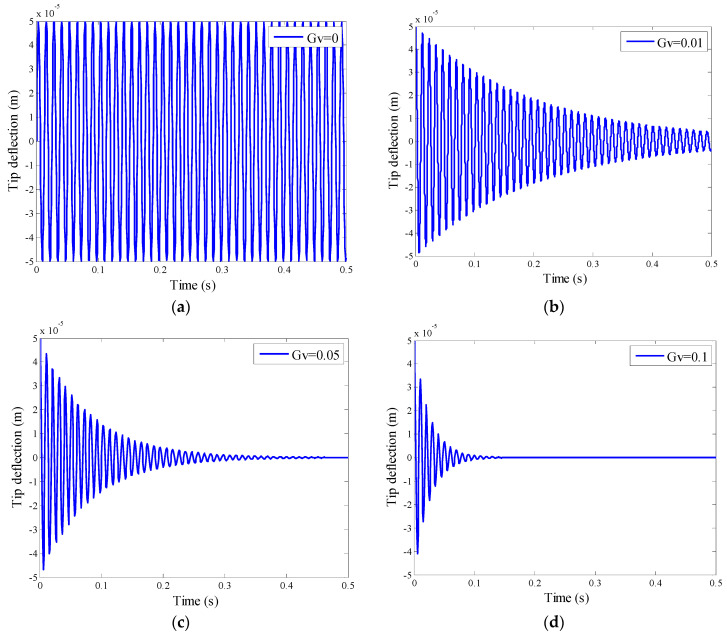
Control effect with different velocity feedback control coefficients. (**a**) Velocity feedback control with $G_{\;v}=0$; (**b**) Velocity feedback control with $G_{\;v}=0.01$; (**c**) Velocity feedback control with $G_{\;v}=0.05$ (**d**) Velocity feedback control with $G_{\;v}=0.1$.

According to the classical speed feedback control method, the sensor and actuator are juxtaposed, so that positive definite damping can be obtained [44,45]. Taking the structure (1) in Figure 3 as the research object, the speed feedback control is adopted, and the active control voltage $V_{e}(t)=G_{\;v}\dot{w}\left(x,y,t\right)$ are taken to study the active control effect. Since the speed feedback control coefficient $G_{\;v}$ will affect the active control damping matrix **C**, thereby changing the system eigenvalues and changing the damping size, the active control effect is different when different speed feedback control coefficients $G_{\;v}$ are taken. Considering the controller with step size of 0.001 s, under the harmonic response $q\left(t\right)=\sin\left(100t\right)N/m^{2}$, four different speed feedback control coefficients $G_{\;v}$ are taken to implement closed-loop control, and the tip deflection responses in the vertical direction of the plate are calculated. The control effect is shown in Figure 5.

Figure 5a shows that when the speed feedback coefficient $G_{\;v}=0$, that is, there is no active control, the end-point deflection of the plate is 0.05 mm.

It can be seen from Figure 5b that the vibration of the plate structure is effectively suppressed within 0.5 s under the condition of the speed feedback coefficient $G_{\;v}=0.01$. The initial maximum deflection has been attenuated from 0.05 mm to 0.01 mm, and the amplitude has decreased by 80%.

Figure 5c shows the effect of active vibration control when the speed feedback coefficient $G_{\;v}=0.05$. It is observed that the initial tip deflection of the plate structure decreases from 0.05 mm to 0.01 mm within 0.2 s. The vibration attenuation time is reduced by 60% compared with Figure 5b. At 0.3 s, the amplitude decays to zero.

It can be seen from Figure 5d that when the speed feedback coefficient is $G_{\;v}=0.1$, the initial tip deflection of the plate decays from 0.05 mm to 0 mm in 0.1 s, the vibration is completely converged. The decay time is reduced by 80% compared to Figure 5b.

It is observed from Figure 5 that the active control of structural vibration can be effectively carried out by using the speed feedback controller. The attenuation time of vibration is greatly shortened under the appropriate value of speed feedback coefficient $G_{\;v}$. The larger the speed feedback value, the faster the vibration of the plate attenuates. By increasing the speed feedback coefficient value, even in very small increments, the vibration suppression effect can be significantly improved. This finding is consistent with the observations and reports of Selim B.A. et al. [46,47]. Obviously, the larger the value of the speed feedback coefficient, the better the active control effect, but a higher coefficient will affect the stability of the control system, resulting in a larger signal-to-noise ratio of the system.

#### 3.2.2. Case II: Control Effect of Different Piezoelectric Patch Locations

**Figure 6:**
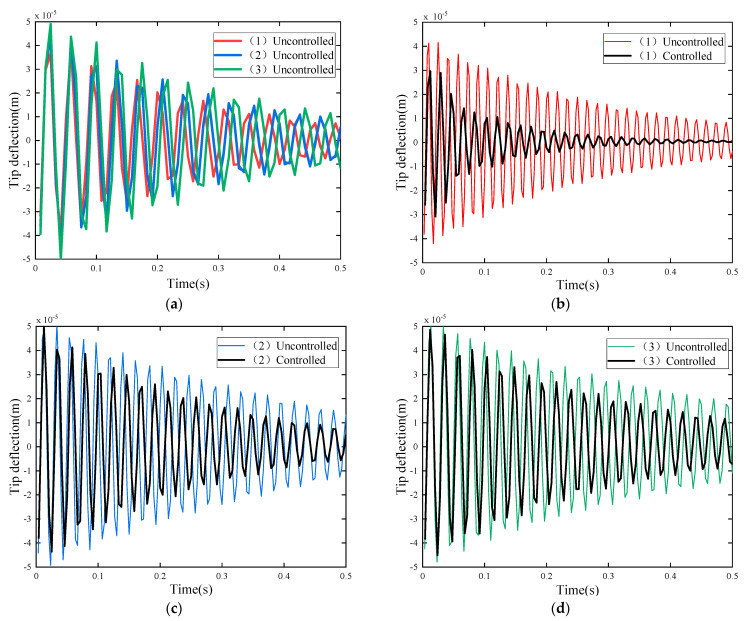
Comparisons of tip deflection attenuation of different structures with or without active control. (**a**) Tip deflection attenuation without active control (**b**) Tip deflection attenuation of structural (1); (**c**) Tip deflection attenuation of structural (2) (**d**) Tip deflection attenuation of structural (3).

In order to study the effect of the piezoelectric patch positions, the vibration active control study is carried out for the structure (1), (2) and (3) shown in Figure 3, respectively. First, the vibration attenuation in the uncontrolled state is investigated. An initial excitation with a uniformly distributed load $q\left(t\right)=40N/m^{2}$ is applied to the structures, respectively. After the excitation is removed, the structure will vibrate freely. The tip deflection attenuations of each structure are shown in Figure 5. Next, the amplitude attenuation of each structure under active vibration control is studied. Take $G_{\;v}=0.05$, other conditions remain unchanged, and the tip deflection attenuations of the three structure with or without active vibration control are shown in Figure 6b–d, respectively.

Figure 6a shows the tip deflection attenuation of the three structures in the uncontrolled state. It can be observed that in the free vibration state, the response convergence of the three structures is not much different. This is because the material coefficients of the three structures are the same, and the difference in structural damping is small. However, the amplitudes are slightly deviated, which is due to the change in the structures after the piezoelectric layer is pasted at different positions. In structure (3), the piezoelectric patch is attached at the free end, the mass of the end increases, and its natural frequency decreases, therefore its amplitude is the largest. In structure (2), the piezoelectric patch is attached at the middle of the plate, and its amplitude is second. In structure (1), the piezoelectric patch is located at the fixed end, and its amplitude is the smallest. The results are in accordance with vibration theory.

Figure 6b shows the tip deflection attenuation of the structure (1) with controlled and uncontrolled station. It can be seen that applying active control can quickly dampen the vibration of the structure. When the piezoelectric sheet is located at the fixed end, the amplitude of the free end is greatly reduced within 0.2 s, and the vibration basically converges within 0.3 s. The vibration suppression effect is remarkable.

Figure 6c shows the tip deflection attenuation of the structure (2) with controlled and uncontrolled station. It is observed the When the piezoelectric sheet is located at the middle, the free end vibration has not converged within 0.5 S. Under the same control, its vibration reduction efficiency is significantly lower than that of structure (1).

Figure 6d shows the tip deflection attenuation of the structure (3) with controlled and uncontrolled station. It can be seen that when the piezoelectric patch is located at the free end, its vibration reduction effect is significantly lower than the former two.

It can be seen from Figure 6 that the placement of the piezoelectric layer in the structure has a great influence on the active vibration control effect. Different engineering problems require different analyses and then the piezoelectric layer is appropriately arranged.

#### 3.2.3. Case II: Control Voltage of Different Structures

**Figure 7:**
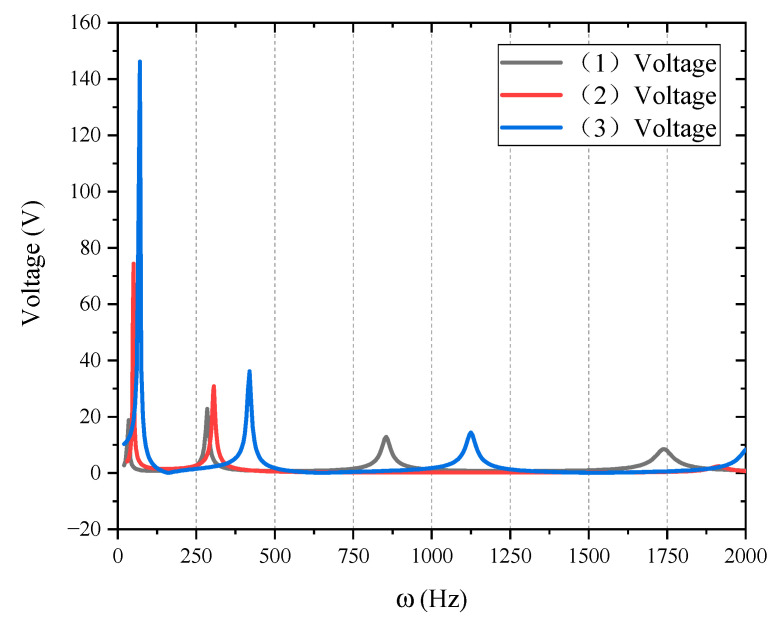
Control voltage of different piezoelectric patch position.

In the active control of the structure, in addition to the control effect, the control cost should also be considered. Control voltage is one of the cost factors. In the active control of structures (1), (2) and (3), although the same speed feedback coefficient is taken, the required control voltages are different. The control voltage value of the piezoelectric layer is shown in Figure 7. Figure 7 shows the frequency domain analysis of the voltage.

From Figure 7, it can be found that the required control voltages of structures (1), (2) and (3) are greatly different when they vibrate at low frequencies (below 150 Hz). Since the active vibration control mainly focuses on the first few modes, the control voltages required by the medium and high frequency above 150 Hz are not much different, and the high-frequency vibration mode has little effect on active control.

**Figure 8:**
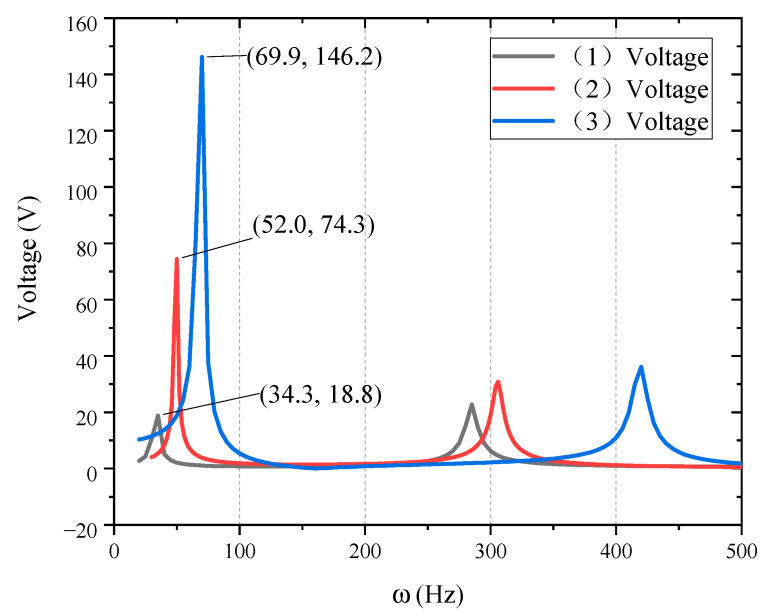
Control voltage of different piezoelectric patch position (Low-frequency zoom).

The low-frequency zoom of Figure 7 is shown in Figure 8.

It can be observed from Figure 8 that the required voltage extreme value of structure (1) is about 18.8 V, for structure (2) it is about 74.4 Vand for structure (3) it is about 146.2 V. In essence, the area between the curve and the coordinate axis represents the power required for active control. The control voltage required by structure (1) is the smallest, structure (2) is the second, and structure (3) is the highest. In terms of amplitude attenuation characteristics and control voltage power, the control effect and control cost of structure (1) are much better than structures (2) and (3), that is, setting piezoelectric patch at the fixed end of the structure has the best active control effect.

## 4. Conclusions

The paper presents static and free vibration analyses and active vibration control of plate sandwiched with piezoelectric sensors and actuators. Based on laminate theory and constitutive equation of piezoelectric material, the active control dynamics equation of the piezoelectric sandwich plate is established by using hypothetical mode method and Rayleigh-Ritz method. A velocity feedback control algorithm is used to adjust the static deflection as well as for active vibration control. Several numerical examples are given to analyze the static deflection, natural vibration mode and active vibration control of piezoelectric sandwich plates with different arrangement schemes of piezoelectric patches. From the present formulation and numerical results, some conclusions are obtained:

1. The method presented in this paper has good accuracy in predicting the static deflection, natural vibration mode of the piezoelectric sandwich plate structures. After adopting the speed feedback algorithm, it can effectively adjust the static deflection as well as for active vibration control of the piezoelectric sandwich plates.
2. The different location of the piezoelectric patch and the different boundary conditions of the piezoelectric sandwich plate have a great influence on the natural vibration modes of the structures. Different input voltages have different effect of on the deflection of the piezoelectric cantilever plate, and they are approximately linear.
3. The velocity feedback coefficient has a great influence on the active vibration control effect of the structure. The larger the value of the speed feedback coefficient, the better the active control effect, but a higher coefficient will affect the stability of the control system, resulting in a larger signal-to-noise ratio of the system.
4. Among the sandwich plate structures with piezoelectric patches arranged in three different positions, the active control study found that the effect of active control is the best when the piezoelectric patches set at the fixed end. After considering the structure of the piezoelectric patch, placing the piezoelectric layer on the fixed end will reduce the natural frequency to a certain extent, the change of the amplitude characteristic is most conducive to the response convergence, and the active control effect is the best. On the contrary, sticking the piezoelectric layer on the free end will as a result, the natural frequency is greatly reduced, and the change of vibration characteristics is the most unfavorable for vibration suppression. In theory, the active control effect of the three structures decreases with regularity.
5. For the active control of the structures with different piezoelectric patches location, although the same speed feedback coefficient is taken, the required control voltages are different.

The model in this paper can be used for the static analysis, dynamic analysis and active control realization of the real piezoelectric sandwich structure. The verification of the position coefficient in this paper provides a reference for the actual layout of piezoelectric layer. In real state, the maximum strain of the structure is the primary consideration for the arrangement of the piezoelectric layer in the active vibration control. Moreover, the model in this paper is limited in dealing the active control problems with complex loads (aerodynamic forces) and complex environments (thermal and wet environments).

The arrangement of the piezoelectric patches affects almost all parameters of the piezoelectric sandwich plate structure including natural vibration modes and active control effects. This paper only studies the active vibration control of three special positions. More piezoelectric optimization design methods and active control algorithms need to be further studied.

## Figures and Tables

**Table 1:** The material parameters of the substrate and piezoelectric patch.

Material Properties	Base Plate	Piezoelectric Layer
Elastic modulus (Gpa)	70	60
Density (kg/m^3^)	2700	7600
Poisson’s ratio	0.35	0.33
Length (mm)	280	40
Width (mm)	40	40
Thickness (mm)	2	2
Piezoelectricconstant (C/m^2^)	—	*e*_31_ = 6.15; *e*_32_ = 6.78
Dielectric Constant (F/m)	—	15 × 10^−9^

**Table 2:** Comparison of the first six natural frequencies of plates with piezoelectric patches at different positions.

Mode	*l_i_/L =* 0			*l_i_/L* = 1/7			*l_i_/L* = 4/7			*l_i_/L* = 7/7		
(Hz)	Present	COMSOL	Δ%	Present	COMSOL	Δ%	Present	COMSOL	Δ%	Present	COMSOL	Δ%
1	21.4	21.6	0.9	28.4	28.8	1.3	18.6	18.1	2.3	11.2	11.5	3.1
2	133.7	137.0	2.4	177.8	183.9	3.4	115.7	116.3	0.6	110.4	111.1	0.6
3	287.1	293.4	2.2	330.3	342.7	1.8	230.2	232.4	0.9	174.7	184.0	5.3
4	378.9	386.1	1.9	486.1	503.1	2.5	355.2	364.1	2.5	218.6	221.3	1.2
5	424.3	440.4	3.8	497.1	508.1	2.2	356.6	364.3	2.2	333.5	356.5	6.9
6	776.5	807.3	3.9	941.9	1022	8.5	776.8	801.7	3.2	676.6	686.3	1.3
7	901.0	870.5	3.5	1032.6	1089.8	5.2	783.9	804.0	2.5	731.9	768.22	4.7
8	1231	1211	1.7	1321.7	1366.6	3.3	1110.3	1161.4	4.4	1217.5	1259.1	3.3

**Table 3:** Comparison of the first six natural frequencies of the plate under different boundary conditions.

Mode	CFSF			SFSF			CFFF			CFCF		
(Hz)	Present	COMSOL	Δ%	Present	COMSOL	Δ%	Present	COMSOL	Δ%	Present	COMSOL	Δ%
1	140.5	140.5	0.0	96.3	96.7	0.4	28.4	28.8	1.3	181.6	181.1	0.3
2	410.7	414.4	0.9	239.9	248.2	3.5	177.8	183.9	3.4	462.4	468.1	1.2
3	676.8	683.6	1.0	570.9	580.6	1.7	330.3	342.8	1.8	682.9	694.9	1.8
4	801.5	819.1	2.1	662.0	667.6	0.8	486.1	503.1	2.5	892.2	907.3	1.7
5	1213.4	1226.7	1.1	969.8	996.8	2.8	497.1	508.1	2.3	1275.1	1324.0	3.8
6	1378.9	1427.2	3.4	1342.1	1401.3	4.4	941.9	1022.0	8.5	1386.9	1421.4	2.5
7	1683.4	1754.1	4.0	1490.8	1583.5	5.9	1032.6	1089.8	5.2	1789.7	1839.8	2.7
8	2100.5	2220.2	5.4	1989.3	2070.7	3.9	1321.7	1366.6	3.3	2108.5	2220.8	5.0

**Table 4:** Tip deflection of the piezoelectric bimorph beam with different input voltages ($\times 10^{-2}\mathrm{mm}$).

Method	Input Voltage			
	50 V	100 V	150 V	200 V
DSG3 [42]	1.727	3.452	5.278	6.904
CS-FEM-DSG3 [42]	1.726	3.451	5.177	6.903
Analytical solution [43]	1.725	3.451	5.175	6.900
Present method	1.725	3.450	5.171	6.899

## Data Availability

Data sharing not applicable.

## Appendix A

$M_{b}=\rho_{b}h_{b}\int_{0}^{a}\int_{0}^{b}WW^{T}dxdy$ . $M_{e}=\rho_{e}h_{e}\int_{0}^{a}\int_{0}^{b}WW^{T}dxdy$ $K_{e}=K_{e1}+K_{e2}+K_{e3}+K_{e4}+K_{e5}+K_{e6}$ $K_{e1}=\frac{Q_{11}^{k}}{3}\left[{\left(\frac{h}{2}+h_{p}\right)}^{3}-{\left(\frac{h}{2}\right)}^{3}\right]\int_{0}^{a}\int_{0}^{b}\frac{\partial^{2}W}{\partial x^{2}}\frac{\partial^{2}W}{\partial x^{2}}dxdy$ $K_{e2}=\frac{2Q_{12}^{k}}{3}\left[{\left(\frac{h}{2}+h_{p}\right)}^{3}-{\left(\frac{h}{2}\right)}^{3}\right]\int_{0}^{a}\int_{0}^{b}\frac{\partial^{2}W}{\partial x^{2}}\frac{\partial^{2}W}{\partial y^{2}}dxdy$ $K_{e3}=\frac{Q_{22}^{k}}{3}\left[{\left(\frac{h}{2}+h_{p}\right)}^{3}-{\left(\frac{h}{2}\right)}^{3}\right]\int_{0}^{a}\int_{0}^{b}\frac{\partial^{2}W}{\partial y^{2}}\frac{\partial^{2}W^{T}}{\partial y^{2}}dxdy$ $K_{e4}=\frac{4Q_{16}^{k}}{3}\left[{\left(\frac{h}{2}+h_{p}\right)}^{3}-{\left(\frac{h}{2}\right)}^{3}\right]\int_{0}^{a}\int_{0}^{b}\frac{\partial^{2}W}{\partial x\partial y}\frac{\partial^{2}W^{T}}{\partial x^{2}}dxdy$ $K_{e5}=\frac{4Q_{26}^{k}}{3}\left[{\left(\frac{h}{2}+h_{p}\right)}^{3}-{\left(\frac{h}{2}\right)}^{3}\right]\int_{0}^{a}\int_{0}^{b}\frac{\partial^{2}W}{\partial x\partial y}\frac{\partial^{2}W^{T}}{\partial y^{2}}dxdy$ $K_{e6}=\frac{4Q_{66}^{k}}{3}\left[{\left(\frac{h}{2}+h_{p}\right)}^{3}-{\left(\frac{h}{2}\right)}^{3}\right]\int_{0}^{a}\int_{0}^{b}\frac{\partial^{2}W}{\partial x\partial y}\frac{\partial^{2}W^{T}}{\partial x\partial y}dxdy$ $K_{d}=\frac{{∍}_{66}}{h_{p}}\int_{0}^{a}\int_{0}^{b}dxdy$ $F=\int_{0}^{a}\int_{0}^{b}qW^{T}dxdy$ $F_{e}=F_{e1}+F_{e2}+F_{e3}$ $F_{e1}=e_{31}(\frac{h_{b}}{2}+h_{e})\int_{0}^{a}\int_{0}^{b}\frac{\partial^{2}W}{\partial x^{2}}dxdy$ $F_{e2}=e_{32}(\frac{h_{b}}{2}+h_{e})\int_{0}^{a}\int_{0}^{b}\frac{\partial^{2}W}{\partial y^{2}}dxdy$ $F_{e3}=2e_{36}(\frac{h_{b}}{2}+h_{e})\int_{0}^{a}\int_{0}^{b}\frac{\partial W}{\partial x\partial y}dxdy$	$K_{b}=K_{b1}+K_{b2}+K_{b3}+K_{b4}+K_{b5}+K_{b6}$ $K_{b1}=\frac{Q_{11}^{k}\sum_{1}^{k}\left(h_{k}^{3}-h_{k-1}^{3}\right)}{3}\int_{0}^{a}\int_{0}^{b}\frac{\partial^{2}W}{\partial x^{2}}\frac{\partial^{2}W^{T}}{\partial x^{2}}dxdy$ $K_{b2}=\frac{2Q_{12}^{k}\sum_{1}^{k}\left(h_{k}^{3}-h_{k-1}^{3}\right)}{3}\int_{0}^{a}\int_{0}^{b}\frac{\partial^{2}W}{\partial x^{2}}\frac{\partial^{2}W^{T}}{\partial y^{2}}dxdy$ $K_{b3}=\frac{Q_{22}^{k}\sum_{1}^{k}\left(h_{k}^{3}-h_{k-1}^{3}\right)}{3}\int_{0}^{a}\int_{0}^{b}\frac{\partial^{2}W}{\partial y^{2}}\frac{\partial^{2}W^{T}}{\partial y^{2}}dxdy$ $K_{b4}=\frac{4Q_{16}^{k}\sum_{1}^{k}\left(h_{k}^{3}-h_{k-1}^{3}\right)}{3}\int_{0}^{a}\int_{0}^{b}\frac{\partial^{2}W}{\partial x\partial y}\frac{\partial^{2}W^{T}}{\partial x^{2}}dxdy$ $K_{b5}=\frac{4Q_{26}^{k}\sum_{1}^{k}\left(h_{k}^{3}-h_{k-1}^{3}\right)}{3}\int_{0}^{a}\int_{0}^{b}\frac{\partial^{2}W}{\partial x\partial y}\frac{\partial^{2}W^{T}}{\partial y^{2}}dxdy$ $K_{b4}=\frac{4Q_{66}^{k}\sum_{1}^{k}\left(h_{k}^{3}-h_{k-1}^{3}\right)}{3}\int_{0}^{a}\int_{0}^{b}\frac{\partial^{2}W}{\partial x\partial y}\frac{\partial^{2}W^{T}}{\partial x\partial y}dxdy$ $K_{c}=K_{c1}+K_{c2}+K_{c3}$ $K_{c1}=\frac{e_{31}}{2h_{b}}\left[{\left(\frac{h}{2}+h_{p}\right)}^{2}-{\left(\frac{h}{2}\right)}^{2}\right]\int_{0}^{a}\int_{0}^{b}\frac{\partial^{2}W}{\partial x^{2}}dxdy$ $K_{c2}=\frac{e_{32}}{2h_{b}}\left[{\left(\frac{h}{2}+h_{p}\right)}^{2}-{\left(\frac{h}{2}\right)}^{2}\right]\int_{0}^{a}\int_{0}^{b}\frac{\partial^{2}W}{\partial y^{2}}dxdy$ $K_{c3}=\frac{e_{36}}{2h_{b}}\left[{\left(\frac{h}{2}+h_{p}\right)}^{2}-{\left(\frac{h}{2}\right)}^{2}\right]\int_{0}^{a}\int_{0}^{b}\frac{\partial W}{\partial y\partial x}dxdy$
